# The Impact of Different Writing Systems on Children’s Spelling Error Profiles: Alphabetic, Akshara, and Hanzi Cases

**DOI:** 10.3389/fpsyg.2020.00870

**Published:** 2020-05-26

**Authors:** Beth A. O’Brien, Malikka Begum Habib Mohamed, Nur Artika Arshad, Nicole Cybil Lim

**Affiliations:** Centre for Research in Child Development, National Institute of Education, Nanyang Technological University, Singapore, Singapore

**Keywords:** spelling acquisition, triple-code theory, error analysis, bilingual children, Southeast Asian scripts

## Abstract

The importance of literacy in academics and the predominantly digital world cannot be understated. The literacy component of writing is less researched than that of reading, even though it holds equal significance for modern success. Spelling is an important aspect of the construct of literacy, and is more difficult to acquire than reading. Previous work on spelling error analysis for English provides insight into the sets of knowledge and cognitive processes required for children to perform the task, and their different strategies across development. However, different sets of skills and strategies may contribute to spelling across types of orthographies. In this study, we extend spelling error analysis to groups of biliterate children learning two scripts, which include English plus either: (a) another Latin-script alphabet with a shallow orthography (Malay); (b) a transparent alphasyllabary using akshara (Tamil); or (c) a non-alphabetic, morphosyllabic script using simplified hanzi characters (Mandarin Chinese). These sets of scripts vary in how speech is mapped to print. We utilized an error coding scheme based on triple-code theory to enumerate the occurrence of phonological, orthographic (graphemic), and morphological (semantic) types of spelling errors across the three language groups. Five hundred and sixty-eight Grade 1, 6-year-old children participated, with 128 English + Malay, 119 English + Tamil, and 321 English + Chinese children in each bilingual group. They completed a spelling to dictation task in their Asian language, with ten words taken from the grade level curriculum per language. Results indicate group differences in the proportions of error types, with more overall errors for Tamil, more phonological errors for Malay, and more irrelevant or non-sense words for Chinese. The implications are that different scripts present different challenges for young learners.

## Introduction

Literacy skills are essential for academic and occupational success. Because of this, the development of literacy proficiency is a key concern for educators, and research into children’s acquisition of said skills is extensive. Reading has received the lion’s share of the research focus, with much less attention paid to spelling. Much of what is known about early spelling skills is based on English and other alphabetic languages, and an extension to a greater variety of writing systems is yet to be established. The focus of this study is on spelling profiles of children learning Asian languages that vary in terms of their writing systems, representing three major types of systems. These include contrastive orthographies of: an alphabetic, Latin-scripted language (Malay); an alphasyllabary script with aksharas (Tamil); and a non-alphabetic, morphosyllabic script using hanzi characters (Chinese).

Following a framework of triple-word-form theory (Berninger et al., 2009; Bahr et al., 2015), we conducted a spelling error type analysis as a means to gain insight into underlying cognitive processes and learning mechanisms related to each of these written languages. A comparison across languages also allows for an accounting of the possible variations of these error types across the scripts. We view the results as an important initial step to developing a broader framework of spelling acquisition that may prove useful in both educational and research contexts, encompassing a wider range of languages.

In the following sections, we first review prominent models of spelling development, then studies of spelling for three different types of written languages: Malay, Tamil, and Chinese. These three languages, along with English, are official languages taught within the context of this study in Singapore. Following the education policy of Singapore, classes are taught in English, but children also learn one of the Asian languages at the same time, based on family ethnicity. Therefore, the current study allows us to examine spelling performance across these divergent language systems for children situated within the same national culture, educational system, and learning side-by-side in the same classrooms.

### Models of Spelling Acquisition – The Case of English

Early studies of English spelling observed the types of spelling errors young learners make (Read, 1971; Gentry, 1982), and gleaned from these a developmental pattern of knowledge that children accumulate with experience. According to this view, children’s knowledge about spelling patterns builds up from learning first an alphabetic principle (that letters represent certain speech sounds), then a pattern principle (that letters go together in certain ways), then a meaning principle (that letter patterns convey meaning in addition to sound) (Johnston et al., 2014). The developmental progression was postulated to follow the types of words encountered over the early grades: from simple syllable structured Anglo-Saxon words that require phonology-to-orthography mapping, to Norman-French words with multi-letter units requiring a consolidation of more complex graphemic units, to Greek and Latin words with stems and affixes to map to meanings (Invernizzi and Hayes, 2004). According to the work of Bear and colleagues, as children develop through spelling phases they may “use but confuse” principles not yet mastered, such as when they write a phonetically plausible but orthographically incorrect spelling, attempting to use letter-sound principles but confusing them (Bear et al., 2016).

Thus, children gradually accumulate different types of knowledge relevant to spelling the types of words they frequently encounter. Other models of spelling proposed that the accumulation of knowledge about spelling patterns does not follow developmental phases, but instead acquired strategies overlap and could be used flexibly at any time in development (Sharp et al., 2008). The emphasis was on the different types of linguistic knowledge required for accurate spelling. Coltheart et al. (2001) focused on lexical and sublexical knowledge in a dual-process model, with the former involving retrieval of whole word spellings from long-term memory, and the latter involving phonology-to-orthography conversion for writing sequences of letters corresponding to the sound sequences in the word. Lexical processing may be predominantly used with irregularly spelled words and sublexical processing for predictable words.

According to triple-word-form theory (Berninger et al., 2009; Garcia et al., 2010; Bahr et al., 2012, 2015) there are three types of linguistic knowledge children need to acquire to spell words correctly. First is phonological knowledge, to perceive, analyze, and identify the spoken word presented, for example, in a word dictation task (e.g., to perceive that the spoken word /sheep/ contains a long vowel and is different from /ship/). Second, there is orthographic knowledge, to produce written symbols (e.g., letters, graphemes, characters) to represent the perceived spoken word (e.g., to write the letter <−e> twice to represent the long vowel /i:/ in /shi:p/). And third is morphological knowledge, to recognize meaningful subunits within a spoken or written word (e.g., to identify that /treehouse/ is composed of the subunits /tree/ and /house/ or that the letter sequence <−ed> in the written word <jumped> represents the past tense inflection of the verb <to jump>). The triple-word-form theory suggests that although all three types of knowledge are necessary to become a proficient speller, children may need to rely to a greater extent on one type of knowledge depending on the word they are aiming to spell. For example, they may show greater reliance on orthographic knowledge to identify the “silent e” in /teibl/ and correctly write <table> instead of <tabl> or <tabel>.

Bahr et al. (2012) developed a coding scheme for spelling error analysis based on the triple-word-form theory. The “Phonological, Orthographic, and Morphological Assessment System (POMAS)” was to categorize spelling errors of English-speaking Primary school students from grade 1 to 9 into phonological, orthographic, and morphological errors. Although initially introduced to explain spelling performance of monolingual English-speaking students, Bahr et al. (2015) adapted the POMAS coding scheme to Spanish to compare the spelling error profiles of Spanish-English bilingual adolescents. Their findings revealed both similarities and differences in the participant’s English and Spanish spelling error profiles indicating a complex interaction of language-common and –specific factors involved in bilingual spelling development. While students committed a similar proportion of phonological errors in both languages, they showed a higher proportion of orthographic errors in Spanish, and the opposite pattern with respect to morphological errors. These results exemplify the potential of the triple-word-form theory as a theoretical framework and more specifically of the POMAS coding scheme as a practical tool to reach a better understanding of students’ spelling performance across different languages. However, it remains unclear if the triple word form theory and the POMAS coding scheme can also be used to investigate spelling error profiles in a greater variety of writing systems, as for example present in many Asian languages.

### Extending Models of Spelling Acquisition to Other Languages

Current research on spelling has extended beyond English, to include a range of more transparent alphabetic orthographies. In studies of Italian and German, early knowledge of word phonology and phonology to orthography correspondence predicted longer-term spelling outcomes years later (Landerl and Wimmer, 2008; Bigozzi et al., 2016). In a study of English, Spanish, Czechia, and Slovak speaking children, Caravolas et al. (2012) showed that spelling outcomes for all languages were similarly explained by letter knowledge, phoneme awareness, and rapid automatized naming. The authors suggested similar required skills for learning to spell across alphabetic languages. In an earlier study, Link and Caramazza (1994) applied the dual-route spelling model to two different writing systems: Arabic and Chinese. While generally compatible with Arabic, an alphabetic system, the model proved inadequate for Chinese, a non-alphabetic system. The model did not sufficiently capture the nature of orthographic representations for either script, though, because it was unclear whether knowledge about letters and diacritics are represented in similar ways within Arabic, while for Chinese the use of subcharacter (sublexical) knowledge for spelling did not appear to be readily distinguishable from character level knowledge (Link and Caramazza, 1994, pp. 283−289). In a more recent computational modeling study, Yang et al. (2013) demonstrated that a triangle model with orthographic, phonological, and semantic meaning-based representations could successfully simulate English and Chinese reading within the same model, suggesting that these three forms of representation underlie a broad range of written languages, at least for reading.

Studies focused more specifically on the diverse languages central to this study are summarized in the next sections. Where available, we review spelling error analysis for the transparent alphabetic system of Malay, alphasyllabaries like Tamil, and the morphosyllabary system of Chinese.

#### Malay Spelling

The Malay written language is a transparent Latin-script alphabet that has mostly 1:1 mappings between letters and their sounds (with only 1 vowel having 2 pronunciations – “e”). The language includes diphthongs and consonant clusters, and affixation is a common feature of words. Although it may be a common assumption that transparent alphabetic languages engender phonologically based strategies for spelling and reading, other forms of knowledge may contribute as well. For example, Rickard Liow and Lee (2004) study with 6−8 year-old Singaporean Malay-speaking children revealed that children with higher spelling ability demonstrated knowledge of suffix spelling even if they mispelled the word stems, whereas weaker spellers did not encode affixes accurately. The authors suggest that even at this young age good spellers may utilize morphological knowledge rather than relying on just single phoneme level correspondences to letters. This contrasts with a finding of young Malay-speakers in Indonesia, whose letter name knowledge was most predictive of spelling errors in word stems, as letter names and sounds are more consistent in Indonesian Malay (Winksel and Lee, 2014). In another study with Singaporean Malay-speaking 5−6 year olds, Jalil and Rickard Liow (2008) found that vowel substitution errors in word and pseudoword spellings were related to invented spellings based on knowledge of familiar words rather than letter-name substitutions or English phoneme-grapheme substitutions.

#### Tamil Spelling

The Tamil written language differs from simple alphabetic systems, because the script units (akshara) involve a spatial configuration that is not a simple linear representation of individual phonemes. Tamil akshara can represent consonants with inherent vowels, short vowels and dipthongs (e.g., the consonant 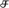/ca/ is formed with a muted stop consonant 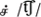 and a short vowel 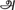 /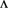/; Aaron and Joshi, 2005). Long vowels can be formed with the addition of diacritical marks where a small change in the writing of the symbol can denote a different sound (e.g., a small curved diacritic mark is included to the short vowel 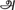 to form the long vowel 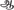; Aaron and Joshi, 2005). This all makes phoneme-to-grapheme conversion more complex than in alphabetic systems. The writing system also differs from strict syllabaries because the akshara can be broken down visually into the consonant and vowel components (Padakannaya and Mohanty, 2004). However, despite the complex orthographic structure, children appear to learn these patterns fairly readily, while still having difficulty representing phonological information even with a relatively transparent script.

Padakannaya and Mohanty (2004) proposed a psycholinguistic model of reading for Brahmi-derived Indian scripts, which involves a dual-route non-lexical and lexical account. Along this line, Nag et al. (2010) found mostly phonologically based, intra-akshara level spelling errors for grade 4−5 students of Kannada – an extensively studied akshara language. Similarly, for Tamil Nag and Narayanan (2019) reported that younger grade 1−3 children in India were able to preserve word length in their spellings, where substitution errors were much more common than the addition or omission of aksharas or intra-akshara phonemic markers. They found orthographic errors were rare, with most errors involving phonological or phonological-orthographic confusions. Older grade 6−12 students in Tamil-medium instruction were found by Aaron and Joshi (2005) to make primarily consonant (retroflex) spelling errors, rather than difficulty with vowels or diacritical marks.

#### Chinese Spelling

The Chinese written language is described as a morphosyllabary, and the script unit of characters (hanzi) are rectangular glyphs that may be a single unit or a complex unit with sub-character components (radicals) that correspond to phonetic or meaning-based representations. Each character (in simplified Chinese) is composed of 1 to 30 different strokes (Chen and Kao, 2002), and most characters in the Chinese orthography include radicals that carry meaning and pronunciation (Ho and Bryant, 1997). An understanding of character structure and radical meaning enhances children’s spelling skill (Lam and McBride, 2018). Shen and Bear (2000) conducted the most extensive descriptive study of children’s spelling errors in Chinese. From a wide sample of primary school students’ Chinese writing samples, they analyzed 15 error types, which included patterns of misspellings (cuo4 zi4) and substitutions (bie2 zi4) at one of the three layers of characters: strokes, radicals, and configurations. They then summarized these as categories of phonological, graphemic, and semantic errors. Phonological error types were initially most prevalent, but with age, graphemic and semantic error types increased in frequency while phonological errors decreased. The authors compared this developmental trend to English spelling development (noted above), from a sound emphasis (alphabetic principle) to shape (pattern principle) to meaning emphasis (meaning principle) across the primary school years.

Tong et al. (2009) utilized a similar coding scheme as Shen and Bear (2000) to examine spelling errors by kindergarteners (6-year-olds) in Hong Kong (though their analysis was at the level of words having two or more characters). In contrast to Shen and Bear (2000), they found the preponderance of errors were meaning-based, morphological types of errors (“lexical, sublexical, single omission”). Orthographic types of errors (“reconfiguration, similar configuration, stroke change”) were the second most frequent, and phonological errors (“homophone and semi-homophone”) were least frequent.

Although an error analysis approach has been usefully applied across the writing systems described above, there has not been a systematic examination across these types of writing systems. As the children in the current study are young bilinguals, we do note that there is evidence of cross-language influence for speaking, listening, reading, and writing as well (Figueredo, 2006). Thus, the findings may be affected by such cross-linguistic influence. An investigation of bilingual spelling patterns is beyond the scope of the current study, but we consider this possible influence in the hypotheses and results. The only bilingual model of spelling of which we are aware, by Tainturier (2019) (BAST), is meant for skilled adult spellers of alphabetic languages and is not a learning model.

#### Current Study

We examine the types of spelling errors for primary grade 1 children learning different scripts according to the triple-word-form model, using 3 error types (phonological, orthographic-graphemic, and morphological) as defined below. We note that these definitions may differ from the manner with which errors have been considered in some previous work (e.g., Shen and Bear, 2000), that followed the “use but confuse” model. In that tradition, errors are defined according to the knowledge children are expected to be using but perhaps not precisely (Bear et al., 2016). We further distinguish the error types here from metalinguistic awareness, which reflects one’s conscious understanding of and ability to analyze and manipulate the sound (phonological) or meaning (morphological) structure of spoken words.

Phonological errors are defined by an incorrect representation of the sounds. This type of error includes the use of an allophone, an omission or addition of phonological elements, which can also include tone, stress, and retroflex (supra segmental). Bahr et al. (2015) refer to these as phonological skeleton elements.

Graphemic-orthographic errors are defined as spelling conveying the same phonology but with incorrect, ambiguous letters. Examples include vowel diagraphs (silent e), consonant diagraphs, letter doubling, flaps, and diacritic marks. Graphemic errors are defined as letter or character reversals, incorrect orientation or misspelling a character with a similar form or omission or addition of strokes.

Morphological-semantic errors are defined as misspelling the target character or word with one that preserves the correct representation of sound but that has a different semantic meaning (e.g., a homophone), or a substitution with a semantically related word. This includes words, or parts of the word, that sound alike but have different meanings. Examples include omission or substitutions of inflections, derivations, and homophones.

Given the variations across scripts, we examined the nature of children’s spelling errors in Malay, Tamil, and Chinese, by addressing the following research questions:

(1) Does the frequency of overall spelling errors differ across the Asian language groups?
  We expect that the orthographic breadth of the scripts will result in more difficulty for the languages with a larger graphemic inventory (Nag, 2017) and more complex units (i.e., Tamil and Chinese).
(2) Is there a difference in the proportion of specific triple-word-form spelling error types across and within the Asian language groups?
  Based on the previous studies reviewed above, we expect that the different scripts will entail different types of spelling errors, with more phonological errors in Tamil, more morphological errors in Chinese, and more phonological and morphological errors in Malay. Alternatively, given the children’s biliterate experience, where they all learn to spell in English at an early age, this may yield a common influence on the prevalence of error types, if spelling strategies transfer between their known languages.
(3) What are the most frequently occurring subtypes of spelling errors for each Asian language group?
  We predict that our findings will follow patterns found for the monolingual and individual language research reported previously, with most frequently phonological error types in Tamil, morphological and orthographic errors in Chinese, and phonological and morphological types in Malay. Alternatively, following linguistic models such as the script dependence hypothesis (Geva and Siegel, 2000) and the transfer facilitation model (Koda, 2007), shared strategies across English and each language may be determined by their typological distance. In this case, Malay would most closely reflect English error types (phonological), while Chinese would deviate with more morphological errors, and Tamil with more orthographic errors.

By understanding the phonological, graphemic-orthographic, and morphological-semantic influences of spelling errors, we may attain a deeper understanding of the basis and source of spelling errors in different languages. This not only provides insight to the rules and strategies that children utilize when spelling words with different scripts, it can also inform instructional activities or inspire pedagogical approaches to facilitate children’s spelling skills acquisition. By determining the domain in which common spelling errors are committed in a certain language, we gain understanding of the unique features each language brings into the language mix.

## Materials and Methods

### Participants

In this study we examined Asian language spelling error profiles of 568 children attending grade 1 of Primary school in Singapore. These children represented a subsample of a larger longitudinal project entitled Singapore Kindergarten Impact Project that aimed to investigate cognitive development from pre- to early primary school (Ng et al., 2014). Findings from overlapping samples in this project are reported in several other publications, such as Yao et al. (2017), Sun et al. (2018), O’Brien et al. (2019), However, this is the first analysis of children’s Asian language spelling errors within this project.

The three different Asian writing systems were chosen due to the typological differences that exist in the multicultural and multiracial environment of Singapore. The demographics of Singapore (Singapore Department of Statistics, 2019, Resident population in Singapore as of June 2018, by ethnic group) are split into 4 main ethnic categorizations of Chinese (74.30%), Malay (13.40%), Indian (9.02%) and Other (3.28%) with official languages – Mandarin, Malay, Tamil and English – corresponding to their ethnic categorisation. The education system in Singapore is bilingual where the main medium of instruction is English and students are taught a second language (Malay, Mandarin, or Tamil), typically based on their ethnic category. Children are exposed to these languages from an early age. The age of onset, quantity, and quality of input received in their home environment in English and one of the above-mentioned Asian languages contributes to their development as simultaneous bilinguals even before entering formal education. Once preschool education commences, their bilingual proficiency is strengthened through daily lessons in English and one of the Asian languages mentioned before. This also includes formal instruction in learning to read and write in both languages. Thus, the sample size and writing systems chosen in this study are considered to reflect the proportion and demographic of the resident population.

The children in this study were native-speakers of English and Chinese (*n* = 321), Malay (*n* = 128), and Tamil (*n* = 119) attending grade 1 of Primary school in Singapore. There were no gender differences in the total number of spelling errors, nor any interaction with the language groups by gender for total errors committed, *F*(2, 561) = 0.33, *p* = n.s. Table 1 presents information on each Asian language group’s background information, including their age, maternal education, and non-verbal reasoning skills, along with home language and literacy environment and bilingual receptive vocabulary skills (see Section “Measures” for details on the sources of this information).

**TABLE 1:** Children’s background information across the three Asian language groups (*N* = 568).

	Chinese (*n* = 321)	Malay (*n* = 128)	Tamil (*n* = 119)	*F-*value	*p-*Value
Age (in months)	80.40(3.76)	81.27(3.70)	81.00(3.92)	2.66	0.071
Maternal education^a^	7.98(2.13)	5.53(2.23)	8.60(1.76)	75.38	<0.001
Non-verbal reasoning skills^b^	16.94(5.02)	14.50(5.19)	13.63(4.87)	22.43	<0.001
Home language environment^c^	0.37(0.44)	0.07(0.47)	0.09(0.55)	27.15	<0.001
Bilingual receptive vocabulary skills^d^	0.15(0.16)	0.09(0.13)	0.17(0.17)	8.20	<0.001
English spelling scores^e^	21.12(4.14)	18.13(4.90)	22.08(4.40)	26.08	<0.001

*^*a*^Measured on a scale from 1 to 11 based on the different educational levels that an adult can achieve in Singapore (for more details see parent questionnaire description). ^*b*^Measured in raw scores. ^*c*^Expressed as an index based on the information reported by parents for English and Chinese or Malay or Tamil home input (see parent questionnaire description). ^*d*^Expressed as the difference between the English and Chinese or Malay or Tamil receptive vocabulary scores obtained by children. ^*e*^Total raw scores Wilkinson and Robertson, 2006. *F*- and *p*-Values are reported from between group ANOVAs.*

While analyses of variance (ANOVAs) revealed that children in all three Asian language groups were of the same age on average, there were significant differences in maternal education, non-verbal reasoning skills, home language and literacy environment, and bilingual receptive vocabulary skills across participant groups. To control for these differences, mothers’ education was used as a matching variable to select a subsample of 120 of these children from the full sample. The same background information on this subsample is described in Table 2.

**TABLE 2:** Subsample’s background information across three Asian language groups (*N* = 120).

	Chinese (*n* = 40)	Malay (*n* = 40)	Tamil (*n* = 40)	*F*-value	*p*-Value
*p* Age (in months)	81.44(3.63)	81.10(4.11)	82.35(3.97)	1.09	0.339
Maternal education	7.03(1.89)	7.03(1.89)	7.03(1.89)	0.00	1.000
Non-verbal reasoning skills	15.33(4.47)	14.74(5.80)	14.33(5.71)	0.342	0.711
Home language environment	0.31(0.50)	0.14(0.49)	0.22(0.54)	0.986	0.376
Bilingual receptive vocabulary skills	0.17(0.18)	0.14(0.24)	0.19(0.12)	0.806	0.449
English spelling scores^a^	19.77(3.23)	19.34(4.77)	20.00(4.52)	0.24	0.79

*^*a*^Total raw scores. Reported measures are the same as in Table 1. *F*- and *p*-values are reported from between group ANOVAs.*

Results from ANOVAs indicated that after controlling for mother’s education, there were no longer differences across Asian language groups in non-verbal reasoning skills, home language and literacy environment nor bilingual receptive vocabulary skills. As explained in the results sections, all analyses were first computed on the full sample of participants that completed the Asian language spelling task (*N* = 568, as in Table 1) and then the same analyses were repeated with the subsample of participants (*N* = 120, as in Table 2) controlling for the impact of the above-mentioned background variables on the results.

### Measures

#### Parent Questionnaire

Parents completed a questionnaire on basic demographics (e.g., age, gender, and ethnicity), and children’s home environment (e.g., parents’ educational qualifications, housing type, household income, amount of time spent with various members of the household on a typical weekday/weekend). Specifically for mother’s education parents were asked to select a number between one and 11 corresponding to one of the following levels of the Singaporean educational level, ranging from completion of: primary school (1), O-level or grade 10 (4), A-level or grade 12 (6), a technical certificate or polytechnic diploma (7, 8), to a bachelor, master, or doctoral degree (9, 10, 11). This information was used as a proxy for children’s socio-economic status (SES). Furthermore, parents were asked to provide information on the quantity of input received by children in each language (i.e., the proportion of exposure to English and to Chinese/Malay/Tamil) per family member, and how much time the child spent with each family member. Based on their responses, we calculated a weighted family wide proportion of language exposure (weighted by the amount of time spent with each family member) for English and for the Asian language (Chinese/Malay/Tamil). Then a relative home language environment index was calculated by subtracting a composite score of language for the Asian language input from that for English. Index scores range from −1 to +1, and positive index scores reflect a stronger home language environment in English than in the Asian language, while a negative index scores indicates the opposite pattern (see Tables 1, 2).

#### Non-verbal Reasoning Skills

The Raven’s Coloured Progressive Matrices (CPM) was used to measure children’s non-verbal reasoning skills (Raven et al., 2004). This task consists of three sets of 12 items of increasing difficulty within each set. Children were asked to select the missing piece between a set of alternatives to complete a matrix. Administration was terminated after four consecutive incorrect responses for each of the three stimuli sets of the task and the total number of correct responses across all three sets was used as the total score of children’s non-verbal responding skills.

#### Bilingual Receptive Vocabulary Skills

For this purpose, the Bilingual Language Assessment Battery (BLAB – Rickard-Liow et al., 2013) was administered. It is a locally developed measure widely used in Singapore (e.g., Yeong and Rickard Liow, 2012), and consists of a spoken word-picture matching with a total of 80 items and three practice trials. The task was rendered on iPads. For each trial, the child listened to an audio-recorded word and selected one of four pictures on the screen that matched the word. Children completed the English version, as well as the Chinese or Malay or Tamil version of the task. Based on children’s scores on the English and Asian language task, an index was computed of the relative bilingual receptive vocabulary skills by subtracting the Chinese/Malay/Tamil score from the English score and dividing this number by the sum of the Asian and English language scores. In this way, positive indices reflected stronger receptive vocabulary skills in English, as compared to Chinese/Malay/Tamil, while negative indices represented the opposite pattern (see Tables 1, 2).

#### Asian Language Spelling

Children in each Asian language group were asked to spell ten words presented through a word dictation task. The examiner first read the item out loud in isolation, then presented it in the context of a sentence, and again dictated the word in isolation. Supplementary Appendix A shows the spelling, IPA transcription, and English translation of the ten words used in each version of the task (Chinese, Malay, and Tamil).

The items were selected from the Singaporean language instruction curricula for Primary school 1 (Ministry of Education [MOE], 2017a,b,c), to obtain an ecologically valid measure of the spelling activities children are exposed to in regular classroom instruction. However, this meant that the items differed naturally in psycholinguistic complexity (e.g., no. of phonemes, no. of graphemes/characters, visual complexity, etc.) across the three Asian language versions, following the characteristic features of each language. Table 3 presents an overview of the psycholinguistic characteristics of the spelling task for each Asian language version and Supplementary Appendix B provides further details for each individual item. The differences in psycholinguistic complexity are inherent to the language-specific characteristics of each of the writing systems, as discussed in the introduction of this report.

**TABLE 3:** Psycholinguistic characteristics of the three Asian language versions of the spelling task.

Psycholinguistic characteristics	Chinese	Malay	Tamil
Phonological characteristics			
No. of phonemes	1 – 3	3 – 9	4 – 11
Phonologically complex items^a^	70%	10%	60%
Graphemic-orthographic characteristics			
No. of graphemes/characters	1	3 − 10	2 – 7
Graphemic complex items^b^	60%	10%	100%
Visual complexity^c^	19−41	10−14	13−26
Morphological-semantic characteristics			
Items with homophones	90%	NA	NA
Morphologically complex items^d^	0%	20%	10%

*^*a*^An item was judged as complex if it contained a dipthong, long vowel, retroflex consonant or consonant cluster and as simple if none of these phonemic units were present. ^*b*^An item was judged as complex if it contained at least one composed grapheme (digraph, composed character, built up akshara) and was otherwise considered simple. ^*c*^Based on multidimensional measure using GraphCom (Chang et al., 2018). ^*d*^An item was judged as complex if it contained at least one pre- or suffix or represented a compound word formed of at least two root words and was otherwise considered simple. NA, Not applicable to this writing system.*

### Procedure

This study derived from the overall longitudinal project (Ng et al., 2014) which received ethical approval from the Nanyang Technological University Institutional Review Board were invited to participate in the overall project when their children were attending Kindergarten 1 (approximately 4 – 5 years of age). Once they signed written consent forms, they were asked to complete the above-mentioned parent questionnaires. Children then participated in a larger battery of tasks in individual testing sessions of approximately 30 to 60 min conducted in a quiet room assigned by the school they were attending. Amongst the overall battery of tasks, we collected information on children’s non-verbal reasoning and bilingual receptive vocabulary skills at Kindergarten 1. In a final wave of data collection at the beginning of Primary school 1 (approximately 6 – 7 years), we administered the Asian language spelling measure that is the focus in this study.

### Data Analysis

As a first step, transcriptions of all children’s responses on the Asian language spelling task were entered into an excel file and the total number of spelling errors committed by each child was tallied. From this, only those children that showed at least one spelling error were included in the analyses. Based on a spelling error coding scheme specifically designed for this study the errors per item per child were classified and summarized. In Table 4 we present an overview of the error categories included in each Asian language coding scheme, and Supplementary Appendix C provides details of the complete coding scheme per language.

**TABLE 4:** Overview of Asian language spelling error coding scheme.

Error category	Chinese	Type	Malay	Type	Tamil	Type
Phonological errors						
Phonetic radical addition, substitution or omission	✓	PC1	NA		NA	
Single vowel substitution	NA		✓	PM1	✓	PT1
Single vowel addition	NA		✓	PM2	✓	PT2
Single vowel omission	NA		✓	PM3	✓	PT3
Dipthong substitution, addition or omission	NA		✓	PM4	✓^NA^	
Short vowel vs. long vowel substitution	NA		NA		✓	PT4
Long vowel vs. short vowel substitution	NA		NA		✓	PT5
Consonant substitution	NA		✓	PM5	✓	PT6
Retroflex consonant substitution	NA		NA		✓	PT7
Consonant addition	NA		✓	PM6	✓	PT8
Consonant omission	NA		✓	PM7	✓	PT9
Similar sounding character/word substitution	✓	PC2	✓	PM8	✓	PT10
Partial reversal of phoneme sequence	NA		✓	PM9	✓	PT11
Graphemic-orthographic errors						
Reconfiguration of characters or components of characters	✓	GC1	NA		NA	
Similar formed or structured character/grapheme substitution	✓	GC2	✓	GM1	✓	GT1
Addition, omission, or protrusion of strokes	✓	GC3	NA	GM2	✓	GT2
Addition, omission or substitution of diacritics	NA		NA		✓	GT3
Morphological-semantic errors						
Substitution of semantically related character/word	✓	MC1	✓	MM1	✓	MT1
Substitution of homophone character/word	✓	MC2	NA		NA	
Morpheme omission (character, pre-/suffix or root)	✓	MC3	✓	MM2	✓	MT2
Other errors						
Substitution by irrelevant word/non-word	✓	OC1	✓	OM1	✓	OT1
No response	✓	OC2	✓	OM2	✓	OT2

*NA, Not applicable to this writing system; ✓, Applicable; NA, Applicable in general to this writing system, but not a possible error with the item set used in this study; PC, PM, and PT, Phonological error for Chinese, Malay, Tamil, respectively; GC, GM, and GT, Graphemic-orthographic error for Chinese, Malay, Tamil, respectively; MC, MM, and MT, Morphological-semantic error for Chinese, Malay, Tamil, respectively; OC, OM, and OT, Other errors for Chinese, Malay, Tamil, respectively.*

Each spelling error was first characterized as a phonological, graphemic-orthographic, semantic-morphological or other spelling error. Then we further categorized these errors into language-specific error types (e.g., vowel addition, etc.). While some of these error subcategories could occur for more than one Asian language (e.g., consonant deletion present in Tamil and Malay coding scheme), others were specific to one of the writing systems (e.g., short versus long vowel substitution is only present in Tamil coding scheme).

Three native-speaking research assistants assigned each spelling error to one of the above-mentioned categories for each Asian language group, respectively. They were previously trained by one of the three authors of this report. In addition, at least 20% of the overall spelling errors were double-scored by the authors of this report and revealed an inter-rater reliability of *K* = 0.79 – 1.00, indicating good agreement between the raters (Feuerman and Miller, 2008) (Chinese: *K* = 0.79, CI 95% [0.72 – 0.86], *p* < 0.001, Malay: *K* = 0.96, CI 95% [0.94 – 0.98], *p* < 0.001, Tamil: *K* = 0.80, 95% CI [0.72 – 0.87], *p* < 0.001). For the Chinese language, consensus was first established between two raters. Subsequently, a third rater completed 20% of the overall scoring and inter-rater reliability was calculated based on this sample. For the Tamil and Malay languages, two raters scored the spelling errors individually and when there was a discrepancy, consensus was reached either through discussion or by consulting with linguistic experts.

## Results

As a first step, in Table 5 we present the descriptive statistics on the total number of spelling errors committed by each Asian language group, as well as on the proportion of different spelling error types (phonological, graphemic-orthographic, morphological-semantic, and others) for the full sample (*N* = 568) that completed the word dictation task. As previously noted in the methods section, there were significant differences between participants’ SES across the three Asian language groups. To avoid the impact of this potentially confounding variable on the analyses, we therefore selected a subsample from each Asian language group using information on mothers’ education as a proxy for SES as a matching variable. Descriptive statistics on the spelling performance of the resulting matched subsample (*N* = 120) are presented in Table 2.

**TABLE 5a:** Average overall errors and proportions of error types for Asian language groups in the full sample (*N* = 568).

Asian language group	Spelling error measure	*M* (*SD*)	*Mdn*	Range	Skewness (*SE*)	Kurtosis (*SE*)
Chinese (*n* = 321)						
	Total errors	7.50 (1.85)	8.00	0.00 − 13.00	−1.02(0.14)	4.30(0.27)
	Proportion of P	0.03 (0.12)	0.00	0.00 − 1.00	6.43(0.14)	45.46(0.27)
	Proportion of G	0.09 (0.13)	0.00	0.00 − 1.00	2.14(0.14)	8.73(0.27)
	Proportion of M	0.09 (0.10)	0.10	0.00 − 0.43	0.90(0.14)	0.09(0.27)
	Proportion of O	0.79 (0.21)	0.83	0.00 − 1.00	−1.42(0.14)	2.84(0.27)
Malay (*n* = 128)						
	Total errors	7.31 (4.38)	8.00	0.00 − 18.00	0.16(0.21)	−0.98(0.43)
	Proportion of P	0.68 (0.24)	0.73	0.00 − 1.00	−1.11(0.21)	1.30(0.43)
	Proportion of G	0.15 (0.18)	0.11	0.00 − 1.00	2.17(0.21)	6.23(0.43)
	Proportion of M	0.06 (0.09)	0.00	0.00 − 0.38	1.23(0.21)	0.60(0.43)
	Proportion of O	0.11 (0.24)	0.00	0.00 − 1.00	2.68(0.37)	7.39(0.73)
Tamil (*n* = 119)						
	Total errors	8.62 (2.23)	9.00	3.00 – 15.00	−0.00(0.22)	0.73(0.44)
	Proportion of P	0.44 (0.31)	0.43	0.00 – 1.00	0.12(0.22)	−1.17(0.44)
	Proportion of G	0.07 (0.78)	0.00	0.00 – 0.30	0.85(0.22)	−0.13(0.44)
	Proportion of M	0.00 (0.03)	0.00	0.00 – 0.25	7.45(0.22)	59.44(0.44)
	Proportion of O	0.49 (0.33)	0.50	0.00 – 1.00	0.02(0.22)	−1.24(0.44)

*P, Phonological errors; G, Graphemic-orthographic errors; M, Morphological-semantic errors; O, Other errors.*

**TABLE 5b:** Average overall errors and proportions of error types for Asian language groups across the subsample (*N* = 120).

Asian language group	Spelling error measure	*M* (*SD*)	*Mdn*	Range	Skewness (*SE*)	Kurtosis (*SE*)
Chinese (*n* = 40)						
	Total errors	7.78 (2.36)	8.00	0.00 – 12.00	−1.65(0.37)	4.40(0.73)
	Proportion of P	0.04 (0.16)	0.00	0.00 – 1.00	5.71(0.37)	34.19(0.73)
	Proportion of G	0.08 (0.11)	0.00	0.00 – 0.33	1.28(0.37)	0.26(0.73)
	Proportion of M	0.08 (0.23)	0.00	0.00 – 0.43	1.49(0.37)	1.54(0.73)
	Proportion of O	0.81 (2.36)	0.89	0.00 – 1.00	−1.50(0.37)	2.65(0.73)
Malay (*n* = 40)						
	Total errors	6.05 (4.43)	4.00	0.00 – 14.00	0.43(0.37)	−1.24(0.73)
	Proportion of P	0.70 (0.24)	0.75	0.00 – 1.00	−1.38(0.37)	1.95(0.73)
	Proportion of G	0.12 (0.12)	0.11	0.00 – 0.38	1.06(0.37)	1.24(0.73)
	Proportion of M	0.07 (0.09)	0.00	0.00 – 0.50	1.38(0.37)	2.05(0.73)
	Proportion of O	0.11 (0.24)	0.00	0.00 – 1.00	2.68(0.37)	7.39(0.73)
Tamil (*n* = 40)						
	Total errors	9.10 (1.98)	9.00	5.00 – 15.00	0.68(0.37)	1.65(0.73)
	Proportion of P	0.40 (0.33)	0.33	0.00 – 1.00	0.44(0.37)	−1.20(0.73)
	Proportion of G	0.07 (0.7)	0.08	0.00 – 0.29	0.82(0.37)	0.31(0.73)
	Proportion of	0.00 (0.02)	0.00	0.00 – 0.13	6.33(0.37)	40.00(0.73)
	Proportion of O	0.53 (0.36)	0.58	0.00 – 1.00	−0.28(0.37)	−1.33(0.73)

*P, Phonological errors; G, Graphemic-orthographic errors; M, Morphological-semantic errors; O, Other errors. Subsample is matched across language groups for SES (mother’s education).*

Descriptive statistics for the full and subsample revealed that the assumptions for parametric statistical analyses were not met for several outcome measures. Therefore, non-parametric analyses were conducted to address each research question.

Each of the research questions mentioned in the introduction section was first addressed by conducting analyses on the full sample dataset (*N* = 568). To compensate for unequal numbers of participants across the three language groups in the full sample (Chinese, *n* = 321; Malay *n* = 128; Tamil *n* = 119), weighted means were used in the analyses. To control for the impact of SES, in a second step the same analyses were repeated on the subsample dataset that included participants matched on mothers’ education (*N* = 120). When comparing the results between the full sample and subsample analyses, there were no overall differences, although effect sizes tended to be larger in the subsample analyses than in the full sample analyses. In the following sections, the set of analyses is presented addressing the research questions.

### Research Question 1

To address the first research question, a Kruskal−Wallis test was computed to identify significant differences in the number of total spelling errors committed by children across the three Asian language groups. The full sample analysis revealed a statistically significant difference across groups, *H*(2, 934) = 45.90, *p* < 0.001, η^2^ = 0.05. *Post hoc* analyses showed that the Tamil language group (*M* = 8.62, *SD* = 2.23, *Mdn* = 9.00) on average committed significantly more spelling errors than the Chinese group (*M* = 7.50, *SD* = 1.85, *Mdn* = 8.00), *H*(1, 678) = 55.00, *p* < 0.001, η^2^ = 0.08, and the Malay group (*M* = 7.31, *SD* = 4.38, *Mdn* = 8.00), *H*(1, 612) = 13.46, *p* < 0.001, η^2^ = 0.02. There was no significant difference between the average number of total spelling errors committed between the Chinese and the Malay language groups, *H*(1, 577) < 0.01, *p* = 0.942, η^2^ < 0.001.

**FIGURE 1:**
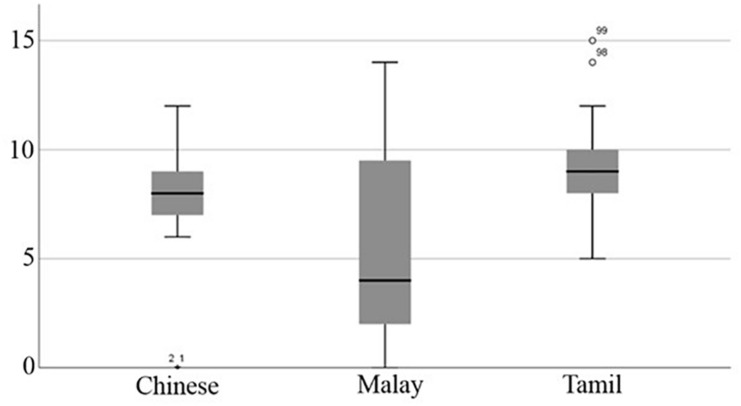
Total number of errors committed by Chinese, Malay, and Tamil Asian language groups in subsample matched for SES (*N* = 120).

Equivalent analysis of the subsample dataset controlling for children’s SES showed the same overall effect as obtained by the full sample analyses. More specifically, there was a significant difference in the average number of total spelling errors committed across the three Asian language groups, *H*(2, 119) = 12.60, *p* = 0.002, η^2^ = 0.11. Again, *post hoc* analyses indicated that Tamil language group (*M* = 9.10, *SD* = 1.98, *Mdn* = 9.00) on average committed significantly more spelling errors than the Chinese group (*M* = 7.78, *SD* = 2.36, *Mdn* = 8.00), *H*(1, 79) = 5.79, *p* < 0.001, η^2^ = 0.07, and the Malay group (*M* = 6.05, *SD* = 4.43, *Mdn* = 4.00), *H*(1, 79) = 10.05, *p* < 0.001, η^2^ = 0.12. There was no significant difference between the average number of total spelling errors committed between the Chinese and the Malay language groups, *H*(1, 79) = 3.43, *p* = 0.060, η^2^ = 0.04. Figure 1 summarizes these results.

### Research Question 2

To address the second research question, the first focus was on investigating potential differences in the proportion of the triple-code types of spelling errors (phonological, graphemic-orthographic, morphological-semantic, and others) committed across Asian language groups. Following Bahr et al. (2015), we assumed potential interrelatedness (ipsitivity) of the four dependent variables measuring different spelling error types expressed in proportions based on the total number of spelling errors committed by each child. Therefore, Kruskal−Wallis tests were computed separately for each spelling error type and a Bonferroni correction was implemented for multiple comparisons to interpret results (adjusted *p* < 0.013 for four comparisons).

**FIGURE 2:**
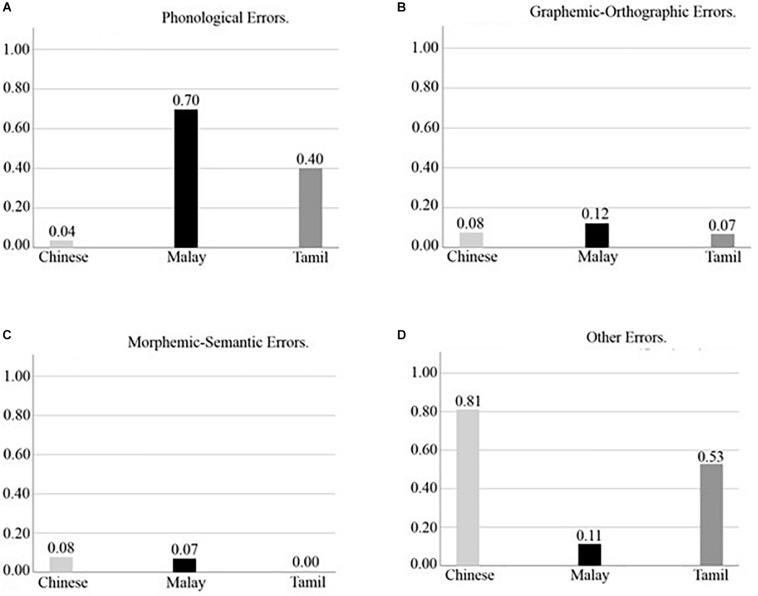
Proportion of Phonological Errors **(A)**, Graphemic-Orthographic Errors **(B)**, Morphemic-Semantic Errors **(C)**, and Other Errors **(D)** in Chinese, Malay, and Tamil Asian language groups in subsample matched for SES (*N* = 120).

Results for the full sample analysis revealed a statistically significant difference in the proportion of phonological errors committed across groups, *H*(2, 934) = 535.85, *p* < 0.001, η^2^ = 0.57. *Post hoc* analyses showed that the Malay language group (*M* = 0.68, *SD* = 0.24, *Mdn* = 0.73) on average evidenced a significantly higher proportion of phonological spelling errors than the Tamil language group (*M* = 0.44, *SD* = 0.31, *Mdn* = 0.43), *H*(1, 612) = 13.46, *p* < 0.001, η^2^ = 0.02 and the Chinese language group (*M* = 0.03, *SD* = 0.12, *Mdn* = 0.00), *H*(1, 577) = 409.96, *p* < 0.001, η^2^ = 0.71. Furthermore, the Tamil language group on average showed a significantly higher proportion of phonological errors than the Chinese language group, *H*(1, 678) = 378.07, *p* < 0.001, η^2^ = 0.56. Analysis on the subsample dataset revealed equivalent results and are detailed in Table 6 and summarized in Figure 2A.

**TABLE 6:** Kruskal Wallis analysis of triple-code errors by language groups in the subsample.

Spelling error measure	Overall results (2, 119)			*Post hoc* analyses								
				Chinese vs. Tamil (1, 79)			Chinese vs. Malay (1, 79)			Malay vs. Tamil (1, 79)		
	*H*	*p*	η ^2^	*H*	*p*	η ^2^	*H*	*p*	η ^2^	*H*	*p*	η ^2^
P	66.54	< 0.001	0.56	41.02	< 0.001	0.52	53.83	< 0.001	0.68	13.84	< 0.001	0.18
G	5.77	0.056	0.05	0.37	0.541	< 0.01	4.45	0.035	0.05	3.71	0.05	0.05
M	20.92	< 0.001	0.18	16.57	< 0.001	0.21	0.04	0.844	< 0.01	20.88	< 0.001	0.26
O	12.60	< 0.001	0.11	14.07	< 0.001	0.18	50.24	< 0.001	0.64	27.82	< 0.001	0.35

*P, Phonological errors; G, Graphemic-orthographic errors; M, Morphological-semantic errors; O, Other errors.*

In relation to the proportion of graphemic-orthographic errors, there were no statistically significant differences across Asian language groups in either the full sample analysis, *H*(2, 678) = 3.91, *p* = 0.048, η^2^ < 0.01, or in the subsample analysis (see Table 6). This means that all three Asian language groups showed similar proportions of graphemic-orthographic errors (see Figure 2B).

For morphological-semantic errors, once again results evidenced significant differences in the proportion of errors of this type committed across the Asian language groups based on the full sample analysis, *H*(2, 678) = 214.46, *p* < 0.001, η^2^ = 0.32. More specifically, the Tamil language group (*M* = 0.00, *SD* = 0.03, *Mdn* = 0.0) showed a significantly lower proportion of morphological-semantic errors than the Chinese language group (*M* = 0.09, *SD* = 0.10, *Mdn* = 0.10), *H*(2, 678) = 214.46, *p* < 0.001, η^2^ = 0.32 and the Malay language group (*M* = 0.06, *SD* = 0.09, *Mdn* = 0.00), *H*(2, 612) = 144.60, *p* < 0.001, η^2^ = 0.24. Furthermore, the Chinese language group showed a significantly higher proportion of morphological-semantic errors than the Malay language group, *H*(2, 577) = 10.23, *p* = 0.001, η^2^ = 0.02. These findings are summarized in Figure 2C and are consistent with the results found in equivalent analysis based on the subsample dataset (see Table 6).

With respect to the proportion of “other” errors, there was also a significant difference across Asian language groups, *H*(2, 934) = 448.02, *p* < 0.001, η^2^ = 0.48. The Chinese language group (*M* = 0.79, *SD* = 0.21, *Mdn* = 0.83) showed a significantly higher proportion of other errors than the Tamil group (*M* = 0.49, *SD* = 0.33, *Mdn* = 0.50), *H*(2, 678) = 144.51, *p* < 0.001, η^2^ = 0.21, and the Malay language group (*M* = 0.11, *SD* = 0.23, *Mdn* = 0.00), *H*(2, 577) = 358.17, *p* < 0.001, η^2^ = 0.62. In addition, results indicated a higher proportion of other errors for the Tamil as compared to the Malay language group, *H*(2, 612) = 213.17, *p* < 0.001, η^2^ = 0.35. Once again, consistent results were found when computing equivalent analyses on the subsample dataset (see Table 6). In Figure 2D the above-mentioned findings are presented.

To address the second part of the second research question, regarding within-group differences, three independent Friedman’s ANOVAs were conducted. These were each done to investigate potential significant differences between the proportion of different spelling error types (phonological, graphemic-orthographic, morphological-semantic, and others) for each group: that is, which of the triple-code error types predominated within each language group?

**FIGURE 3:**
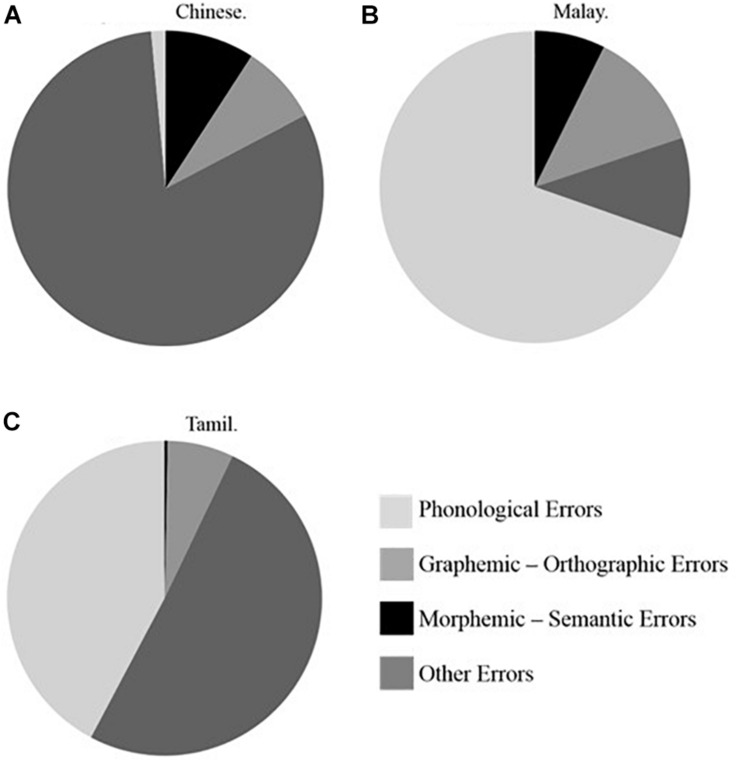
Proportion of Phonological Errors, Graphemic-Orthographic Errors, Morphemic-Semantic Errors, and Other Errors in Chinese **(A)**, Malay **(B)**, and Tamil **(C)** Asian language groups in subsample matched for SES (*N* = 120).

First, for the Chinese language group there was an overall significant difference between the proportion of spelling error types in the full sample analysis, *F*(3, 321) = 699.20, *p* < 0.001. The highest proportion of errors was found for the “other” category of errors (*M* = 0.79, *SD* = 0.21, *Mdn* = 0.83), followed by morphological-semantic errors (*M* = 0.09, *SD* = 0.10, *Mdn* = 0.10) and graphemic-orthographic errors (*M* = 0.09, *SD* = 0.13, *Mdn* = 0.00), and finally phonological errors last (*M* = 0.03, *SD* = 0.12, *Mdn* = 0.00). Analysis of the subsample for the Chinese language group replicated these results, *F*(3, 39) = 89.59, *p* < 0.001 and indicated a significantly higher proportion of “other” errors as compared to phonological, graphemic-orthographic, and morphological-semantic errors, as revealed by *post hoc* analyses, *p* < 0.001. Figure 3A illustrates these results.

Second, for the Malay language group there was also an overall significant difference between the proportion of error types children committed, *F*(3, 127) = 204.88, *p* < 0.001. Phonological errors showed the highest proportion (*M* = 0.68, *SD* = 0.24, *Mdn* = 0.73), followed by graphemic-orthographic errors (*M* = 0.15, *SD* = 0.18, *Mdn* = 0.11), other errors (*M* = 0.11, *SD* = 0.23, *Mdn* = 0.00), and morphological errors (*M* = 0.06, *SD* = 0.09, *Mdn* = 0.00). Equivalent subsample analysis also showed a significant effect of error type, *F*(3, 39) = 66.94, *p* < 0.001. *Post hoc* analyses revealed significant differences between the proportion of phonological and morphological-semantic and other errors on one hand, and between other and morphological-semantic errors and graphemic-orthographic errors on the other hand, *p* < 0.001. These results are summarized in Figure 3B.

Finally, for the Tamil group results once again showed a significant overall difference between the proportion of different error types committed by children, *F*(2, 119) = 216.42, *p* < 0.001. “Other” errors occurred in the highest proportion (*M* = 0.49, *SD* = 0.33, *Mdn* = 0.50), followed by phonological (*M* = 0.44, *SD* = 0.31, *Mdn* = 0.43), graphemic-orthographic (*M* = 0.07, *SD* = 0.78, *Mdn* = 0.00) and finally, morphological-semantic (*M* = 0.00, *SD* = 0.03, *Mdn* = 0.00). Parallel subsample analysis confirmed the overall difference between error types, *F*(3, 39) = 66.94, *p* < 0.001. In addition, *post hoc* analyses indicated a significantly higher proportion of phonological errors and other errors as compared to graphemic-orthographic and morphological-semantic errors, respectively, *p* < 0.001. Figure 3C reflects these results.

### Research Question 3

In relation to the third research question, we aimed to further characterize the specific subtypes of spelling errors committed within each broader error category (phonological, graphemic-orthographic, morphological-semantic, and others) that emerged from the coding scheme used in this study (see Section “Methods” and Supplementary Appendix C). To this end, we calculated the frequencies of each subtype of spelling error committed by children in each Asian language group. In Tables 7a–c information is presented on the three most frequently occurring subtypes of errors for each broader error category. These data were computed across the full sample.

**TABLE 7a:** Most frequently occurring subtypes of spelling errors within triple-code categories for Chinese.

Spelling error measure	Total *n*	Rank 1	Rank 2	Rank 3
Other errors	1908	Substitution by irrelevant word/non-word (OC1 − *n* = 1304)	No response (OC2 − *n* = 604)	
Morphological-semantic	243	Substitution of homophone (MC2 − *n* = 108)	Morpheme omission (MC3 − *n* = 79)	Substitution of semantically related character (MC1 − *n* = 56)
Graphemic-orthographic	219	Addition, omission or protrusion of strokes (GC3 − *n* = 171)	Similar formed or structured character substitution (GC2 − *n* = 39)	Reconfiguration of characters or components of characters (GC1 − *n* = 9)
Phonological errors	39	Phonetic radical addition, substitution or omission (PC1 − *n* = 39)	Similar sounding character/word substitution (PC2 – *n* = 0)	

**n*, number of errors across the full sample of Chinese-speaking participants.*

**TABLE 7b:** Most frequently occurring subtypes of spelling errors within triple-code categories for Malay.

Spelling error measure	Total *n*	Rank 1	Rank 2	Rank 3
Other errors	234	Substitution by irrelevant word/non-word (OM1 − *n* = 185)	Non-response (OM2 – *n* = 49)	
Morphological-semantic	85	Morpheme Omission (MM2 – *n* = 55)	Substitution of semantically related character/word (MM1 − *n* = 30)	
Graphemic-orthographic	102	Similar formed or structured grapheme substitution (GM1 − *n* = 102)		
Phonological errors	375	Single vowel substitution (PM1 − *n* = 186)	Single vowel omission (PM3 − *n* = 119)	Consonant omission (PM7 − *n* = 70)

**n*, number of errors across the full sample of Malay-speaking participants.*

**TABLE 7c:** Most frequently occurring subtypes of spelling errors within triple-code categories for Tamil.

Spelling error measure	Total *n*	Rank 1	Rank 2	Rank 3
Other errors	474	Substitution by irrelevant word/non-word (OT1 − *n* = 250)	No response (OT2 − *n* = 224)	
Phonological errors	471	Consonant substitution (PT6 − *n* = 118)	Retroflex consonant substitution (PT7 − *n* = 109)	Similar sounding word substitution (PT10 − *n* = 50)
Graphemic-orthographic	75	Similar formed or structured grapheme substitution (GT1 − *n* = 71)	Addition, omission or substitution of diacritics (GT3 − *n* = 2)	Addition, omission or protrusion of strokes (GT2 − *n* = 2)
Morphological-semantic	4	Morpheme omission (MT2 − *n* = 4)	Substitution of semantically related word (MT1 – *n* = 0)	

**n*, number of errors across the full sample of Tamil-speaking participants.*

## Discussion

With the aim of broadening frameworks of spelling acquisition, we examined children’s performance in diverse types of scripts using a traditional approach of error type analysis. From a large group of children in primary grade 1 learning an Asian alphabetic script, akshara script or hanzi script, we asked whether the nature of spelling errors differed across scripts, and whether there are commonalities. By identifying script-general and script-specific features of spelling skills in this systematic investigation across the writing systems, this study contributes to a broader framework of spelling acquisition which should prove useful in a range of educational and research contexts.

### Overall Errors Across Language Groups

The first finding indicated that of the three groups we examined, those spelling Tamil words made more errors overall than the other groups. This was true within the whole sample, and the effect was even larger when SES was controlled within the matched subsample. Several reasons could explain this result, including the large orthographic inventory for Tamil, where children must learn the 247 graphemic representations or aksharas. This was in line with our expectation that orthographic breadth would yield more difficulties with spelling. While this challenging inventory size would apply even moreso to children learning Chinese characters, for Tamil there are both visually confusable and phonetically confusable aksharas which require attention to fine details of the complex symbols. That is, while the Chinese characters had a higher range of visual complexity compared with the Tamil aksharas (based on Chang et al., 2018), all items in the Tamil list were considered graphemically complex compared with 60% of the Chinese items being graphemically complex (refer to Table 3). Also, the mapping system from speech to print is more arbitrary for Chinese, whereas there is a systematic relationship for aksharas. As such, children first learning Tamil may begin to understand this mapping system and therefore may be more able to attempt to spell words which they are unsure of. With Chinese, young learners may not have a systematic knowledge to draw upon when attempting to write unknown characters, which may explain a large proportion of blank responses or random guesses when they are unsure of how to spell the Chinese characters. Further, even though the relation of spelling to sound may be very systematic for Tamil, the relation is also complex, as noted by Nag and Narayanan (2019), where both syllable and phoneme levels are represented within words. Making sense of this dual-level representation of phonology in writing may take time for children to acquire.

### Specific Types of Errors Across Language Groups

Inspection of the broad types of errors that contributed to children’s performance revealed differences across the scripts when a triple-code framework was considered. Examining phonological, morphological, and orthographic-graphemic errors separately, the language groups were only similar in the proportion of the latter type. All three groups differed in the amount of phonological types of errors, where these were most prevalent in Malay spelling and least prevalent in Chinese spelling, with Tamil spelling coming in between the two. Given the alphabetic structure of Malay, as well as Tamil to some degree, these errors may not be surprising. The Tamil and Chinese findings fit with previous spelling studies. Nag et al. (2010), Nag and Narayanan (2019) found most akshara-based spelling errors to be of a phonological nature. Further, Tong et al. (2009) found in their Hong Kong sample of kindergarteners that phonological errors were the most rare type in children’s character writing. These errors were also very rare for the current sample of Chinese speakers.

Considering morphological types of errors, these were very rare in Tamil spellings. Both the Chinese and Malay language groups made more of these types of errors than did the Tamil group. Even though these types of errors were not that frequent in Chinese and Malay, the occurrence rates were similar across these two groups. It was suggested in earlier studies by Rickard Liow and Lee (2004) that Singaporean children learning to spell in Malay tended to use morphological knowledge. As well, Tong et al. (2009) found that morphologically based errors predominated in Chinese writing by young children. The set of results followed previous monolingual findings for each of the three languages, according to phonological, orthographic and morphological error types, and did not support the alternative hypothesis that cross-linguistic influence from learning English would result in similar types of errors across the Asian languages.

Finally, the “other” category of errors, which included blank responses or substitutions of unrelated words (otherwise considered random guesses), also differed in occurrence rates between all the language groups. These were by far most common for Chinese writing, with Tamil writing coming in second highest for this category, and were more rare for Malay writing. The ability to sound out words and the use of a very familiar, constrained alphabet may have meant that Malay spellers are better positioned to make more accurate or plausible attempts at unknown spellings with either phonologically or morphologically close approximations.

### Prevalent Error Types per Language

Zooming in within each language group, there were some expectations in terms of the types of errors that children may be most susceptible to given the nature of the various scripts. For Chinese, it was expected that morphologically based errors would be most frequent, as in Tong et al. (2009), and graphemic-orthographic errors would be second highest in proportion. These two error types were statistically equivalent in their rates in the current study, but they were much less common than the top “other” error type. As noted above, some of these “other” errors included blank, null responses, but most “other” errors (68% of them) involved writing an unrelated word or a made-up, illegal character (Table 7a). Thus, children often attempted to write something even when they did not know how to represent the dictated word. Across all errors, morphological-semantically based homophone substitutions accounted for only 4.5% of all errors committed, while sub-character omissions or substitutions related to morphemes accounted for about 5.6% of overall errors. This indicates that neither whole character nor sub-character knowledge related to meaning played a major role in children’s spelling at this stage in the current sample. More frequent in terms of overall errors were stroke mistakes (about 7%) where strokes were either omitted, added or inaccurate, while at the same time configuration errors were almost non-existent. This suggests children had general orthographic knowledge with regard to the overall orientation and how components are represented in characters, but character-specific knowledge about stroke components is still developing. Likewise, phonological information did not play a role in children’s spelling attempts, with just over 1% of overall errors involving phonetic radical additions, omissions or substitutions (Table 7a).

For Malay spelling, although similar transparent alphabetic languages using the Latin script suggest a strong reliance on phonological knowledge for decoding and encoding, previous research in Singapore indicated the importance of morphological knowledge. Jalil and Rickard Liow (2008) found that children’s vowel substitutions came more from familiar words that were similar to the target word, rather than phonemic substitutions. Rickard Liow and Lee (2004) also suggested a stronger reliance on morphological knowledge by young Malay spellers. According to the present sample, phonologically based errors were predominant, with graphemic-orthographic errors second most common. Both of these were greater than the morphological and other error types. Phonological errors involved mostly vowel substitutions or omissions (23% and 15% of overall errors, respectively). The other types of errors, where irrelevant or non-words were written, accounted for 23% of overall errors. And graphemic-orthographic errors involving letter reversals accounted for 12% of overall errors. Thus, when attempting to spell unfamiliar words in Malay, children tend to write something, and they tend to approximate the actual word rather than writing non-sense words (only 6% of errors were non-response, blanks). Their approximations involve confusions mainly about how vowel sounds are represented, with some still confusing letters with reversed forms (e.g., “b” for “d”).

As for Tamil, we expected that vowels and their diacritical representations would prove most difficult, following findings by Nag and Narayanan (2019), and that these would therefore include phonologically based errors. We found that most children’s spelling errors involved either other types (49%) or phonological types (44%) with almost no morphological errors. Out of all the errors, 24% included wrong words or non-word attempts, while 22% were left blank. Another 22% of all errors were due to substitutions of consonants. Thus, the present findings followed from Nag and Narayanan (2019) in that many errors were phonologically based, but in this sample they were more similar to the older learners in Aaron and Joshi (2005), who also found more consonant than vowel spelling errors. On the other hand, there were only few graphemic-orthographic errors overall (7% of all errors), suggesting children quickly develop an understanding of how the akshara are formed, similar to Nag and Narayanan (2019), but they require a longer period of time to distinguish between representation of consonants within akshara.

Overall, the present findings suggest major differences in how children who are in the beginning stages of literacy acquisition encode words, in this case in their other language (besides English). Of the triple-code representations for words – i.e., sound, shape and meaning-based codes (phonological, orthographic, and morphological-semantic) – these do not appear to follow a universal developmental trend, as suggested by Shen and Bear (2000). Rather, the multiple knowledge sources from the triple codes may developmentally overlap, as suggested by Bahr et al. (2009). Thus, the use of different representational codes can be dynamically applied to the situation or specific item to be encoded. This means children may need to rely on different types of knowledge depending on the word they are trying to spell – they could adjust to relying to a greater extent on their phonological, orthographic or morphological knowledge. Furthermore, the specifics of the script in use and the constraints of that script may require use of a specific representational code to a greater or lesser extent.

Thus, rather than a universal set of stages that children progress through on their way to becoming proficient spellers, children more likely accumulate knowledge of the orthographic patterns of their script as well as the phonological and morphological information these encrypt (e.g., Sharp et al., 2008). Treiman and Kessler (2014) theory of Integration of Multiple Patterns (IMP) accounts for how children apply multiple patterns for spelling even within the same word. They contend that what is learned includes both general patterns and word specific patterns of spelling, and that these are accumulated with experience that may involve both implicit learning (statistical learning) of properties of the language, as well as explicit instruction in terms of general rules. Learned patterns also include how they relate to linguistic features of the spoken language. Similar to this, Nag and Narayanan (2019) find overlap in children’s mastering of multiple orthographic features of Tamil, where some features take a longer span of time to master especially vowel markers like diacritics.

Regarding universal aspects of spelling, Verhoeven and Perfetti (2017) specify that all writing systems utilize a graphical script with orthographic principles for how it maps to different linguistic units: phonemes, morphemes, syllables. As learners gain experience with specific words, they may become able to retrieve whole words (lexical access) without fully computing the mapping principle. But initially, decoding or encoding words is computationally driven, such as through morphological deconstruction or phonological recoding of sub-character/sub-word units. Where languages differ is how these computations are carried out – either via activation of only phonology, or some combination of phonology and other codes like morphology. In the present study, we tried to gain a glimpse through children’s spelling patterns (and error types) into the types of representations that they activate to spell words in their various scripts.

### Limitations

The approach we took to studying early spelling acquisition across different writing systems has some limitations and may require further study in order to generalize the results. First, for the spelling error analysis, we used an approach whereby children were asked to spell lists of words derived from their class curricula. While this provided ecological validity and yielded sets of items that typify the types of linguistic material they experience in a learning environment, it did not allow us to compare performance across a more uniform set of items. Experimentally controlled lists of words that are equalized across the scripts in terms of psycholinguistic properties would allow a more balanced assessment of the extent to which the triple codes are applied to the task of spelling. For example, there were few instances of complex words in the Malay list, meaning morphological complexity was limited. Also, there were only two types of phonologically based errors for Chinese character writing. Future studies could attempt to equate the numbers of these items even if they are less frequent in some languages.

Another drawback is that we only examined children’s performance cross-sectionally at one point in time, so it is difficult to make judgements about the direction of learning the underlying principles for spelling. For example, phonological errors may mean they are attempting to represent the sounds correctly, but fall short of accurately doing so. We considered that if children are making errors in some realm, e.g., phonology, then they have not yet mastered the principles of their script based on that code. In other words, children may make many phonological errors and few orthographic errors, because, we would assume, they have mastered orthographic principles but not yet phonology-orthography principles. To disentangle these assumptions, a longitudinal examination of the growth or stability of accurate features would need to be conducted.

Finally, the current sample includes a set of children in a specific multilingual context, with all experiencing English as the school medium of instruction, and many learning an Asian language as a second or heritage language. Therefore, cross-language influence could come into play and there may be differences in how children process the Asian language scripts compared to those living abroad.

## Conclusion

Despite the shortcomings, this study may contribute some theoretical and practical implications. That is, comparison of children within the same classrooms learning different Asian scripts allows us to consider what strategies and challenges are present for all scripts, and which may be script-specific. What do children “pick-up” on early in their literacy learning of each script? Following from Treiman and Kessler (2014) IMP model, this provides information about the types of features children implicitly learn from their experience with their language, and the features that prove problematic (e.g., phonological for Malay and Tamil). Across all the groups, graphemic-orthographic errors were similarly rare, indicating that the visual-orthographic code may be picked up early on. The number of “other” errors for Chinese and Tamil suggests that explicit teaching of general patterns regarding how the orthographic patterns relate to spoken language may be in order. As Treiman and Kessler (2014) explain, the patterns in writing systems allow children to make generalizations across words, and instructional supports may aid in such generalizations. They consider that children can benefit from feedback not only about how their misspellings are wrong, but why they may be wrong. By highlighting triple-code theory, we can consider how to focus such feedback on the relevant types of errors children tend to make when learning different types of scripts.

## Data Availability Statement

The datasets generated for this study are available on request to the corresponding author.

## Ethics Statement

The studies involving human participants were reviewed and approved by NTU Institutional Review Board. Written informed consent to participate in this study was provided by the participants’ legal guardian.

## Author Contributions

BO’B contributed to conception of the work, interpretation of data, and drafting the work for intellectual content. MH contributed to design of the work, data acquisition, interpretation, and revising work. NA contributed to data acquisition, interpretation, and revising work. NL contributed to data analysis, interpretation, and revising work.

## Conflict of Interest

The authors declare that the research was conducted in the absence of any commercial or financial relationships that could be construed as a potential conflict of interest.
