# Supplementary material for: The Impact of Different Writing Systems on Children’s Spelling Error Profiles: Alphabetic, Akshara, and Hanzi Cases
Source: Front Psychol. 2020 May 26;11:870. doi: 10.3389/fpsyg.2020.00870 (PMC7264392; doi:10.3389/fpsyg.2020.00870)
Supplement: Supplementary file 1 [file Data_Sheet_1.docx]

**APPENDIX A**

**Items used in Asian language spelling tests**

| Item No. | Chinese | | | Malay | | | Tamil | | |
| --- | --- | --- | --- | --- | --- | --- | --- | --- | --- |
|  | Spelling | IPA transcription | English translation | Spelling | IPA transcription | English translation | Spelling | IPA transcription | English translation |
| 1 | 五 | /uː^3^/ | five | dan | /dan/ | and | பல | /pala/ | many |
| 2 | 个 | /kɤ^5^/ | one (qualifier) | ibu | /ibu/ | mother | படம் | /pad̪am/ | picture |
| 3 | 早 | /tsau^3^/ | morning | mata | /mata/ | eyes | கரம் | /kaɾam/ | hand |
| 4 | 门 | /mən^2^/ | door | guru | /guru/ | teacher | பழம் | /paɺam/ | fruit |
| 5 | 写 | /ɕeː^3^/ | write | dapur | /dapur/ | kitchen | தாய் | /t̪a:i/ | mother |
| 6 | 坐 | /tsʷoː^4^/ | sit | pantas | /pantas/ | fast | கணினி | /gaɳini/ | computer |
| 7 | 衣 | /[iː^1^](https://easypronunciation.com/)/ | clothes | tidur | /tidur/ | sleep | இன்பம் | /inbam/ | joy |
| 8 | 妈 | /maː^1^/ | mother | membaca | /məmbat͡ʃa/ | reading | விமானம் | /ʋima:nam/ | flight |
| 9 | 老 | /lau^3^/ | old | permainan | /pərmai͡nan/ | game | நாற்காலி | /na:ɺka:li/ | chair |
| 10 | 玩 | /wæn^2^/ | play | televisyen | /televiʃen/ | television | உடற்பயிற்சி | /ud̪eɺpajiɺt͡ʃi/ | exercise |

**APPENDIX B**

**Psycholinguistic characteristics of items in the Asian language spelling test**

**Chinese**

| Item No. | Item | | | Phonological characteristics | | Graphemic-orthographic characteristics | | | Morphological-semantic characteristics | |
| --- | --- | --- | --- | --- | --- | --- | --- | --- | --- | --- |
|  | Spelling | IPA transcription | English translation | No. of phonemes | Phonological complexity^a^ | No. of graphemes/ characters | Graphemic complexity^b^ | Visual complexity | Presence of homophones | Morphological complexity^c^ |
| 1 | 五 | /uː^3^/ | five | 1 | complex | 1 | simple | 25 | yes | simple |
| 2 | 个 | /kɤ^5^/ | one (qualifier) | 2 | simple | 1 | complex | 19 | yes | simple |
| 3 | 早 | /tsau^3^/ | morning | 3 | complex | 1 | complex | 32 | yes | simple |
| 4 | 门 | /mən^2^/ | door | 3 | simple | 1 | simple | 21 | yes | simple |
| 5 | 写 | /ɕeː^3^/ | write | 2 | complex | 1 | complex | 33 | no | simple |
| 6 | 坐 | /tsʷoː^4^/ | sit | 2 | complex | 1 | complex | 35 | yes | simple |
| 7 | 衣 | /[iː^1^](https://easypronunciation.com/)/ | clothes | 1 | complex | 1 | simple | 34 | yes | simple |
| 8 | 妈 | /maː^1^/ | mother | 2 | complex | 1 | complex | 41 | yes | simple |
| 9 | 老 | /lau^3^/ | old | 3 | complex | 1 | simple | 32 | yes | simple |
| 10 | 玩 | /wæn^2^/ | play | 3 | simple | 1 | complex | 39 | yes | simple |

*Note.* ^a^An item was judged as complex if it contained a diphthong, long vowel, retroflex consonant or consonant cluster and as simple if none of these phonemic

units were present. ^b^An item was judged as complex if it contained at least one composed grapheme (composed character) and was otherwise considered simple. ^c^An item was judged as complex if it contained at least one pre- or suffix or represented a compound word formed of at least two root words and was otherwise considered simple.

**Malay**

| Item No. | Item | | | Phonological characteristics | | Graphemic-orthographic characteristics | | | Morphological-semantic characteristics | |
| --- | --- | --- | --- | --- | --- | --- | --- | --- | --- | --- |
|  | Spelling | IPA transcription | English translation | No. of phonemes | Phonological complexity^a^ | No. of graphemes/ characters | Graphemic complexity^b^ | Visual complexity | Homophones | Morphological complexity^c^ |
| 1 | dan | /dan/ | and | 3 | simple | 3 | simple | 11 | no | simple |
| 2 | ibu | /ibu/ | mother | 3 | simple | 3 | simple | 10 | no | simple |
| 3 | mata | /mata/ | eyes | 4 | simple | 4 | simple | 12 | no | simple |
| 4 | guru | /guru/ | teacher | 4 | simple | 4 | simple | 11 | no | simple |
| 5 | dapur | /dapur/ | kitchen | 5 | simple | 5 | simple | 11 | no | simple |
| 6 | pantas | /pantas/ | fast | 6 | simple | 6 | simple | 11 | no | simple |
| 7 | tidur | /tidur/ | sleep | 5 | simple | 5 | simple | 10 | no | simple |
| 8 | membaca | /məmbat͡ʃa/ | reading | 7 | simple | 7 | simple | 14 | no | complex |
| 9 | permainan | /pərmai͡nan | game | 8 | complex | 9 | simple | 12 | no | complex |
| 10 | televisyen | /televiʃen/ | television | 9 | simple | 10 | complex | 10 | no | simple |

*Note.* ^a^An item was judged as complex if it contained a diphthong, long vowel, retroflex consonant or consonant cluster and as simple if none of these phonemic

units were present. ^b^An item was judged as complex if it contained at least one composed grapheme (digraph) and was otherwise considered simple. ^c^An item was judged as complex if it contained at least one pre- or suffix or represented a compound word formed of at least two root words and was otherwise considered simple.

**Tamil**

| Item No. | Item | | | Phonological characteristics | | Graphemic-orthographic characteristics | | | Morphological-semantic characteristics | |
| --- | --- | --- | --- | --- | --- | --- | --- | --- | --- | --- |
|  | Spelling | IPA transcription | English translation | No. of phonemes | Phonological complexity^a^ | No. of graphemes/ characters | Graphemic complexity^b^ | Visual complexity | Homophones | Morphological complexity^c^ |
| 1 | பல | /pala/ | many | 4 | simple | 2 | complex | 16 | no | simple |
| 2 | படம் | /pad̪am/ | picture | 5 | simple | 3 | complex | 13 | no | simple |
| 3 | கரம் | /kaɾam/ | hand | 5 | simple | 3 | complex | 18 | no | simple |
| 4 | பழம் | /paɺam/ | fruit | 5 | complex | 3 | complex | 18 | no | simple |
| 5 | தாய் | /t̪a:i/ | mother | 3 | complex | 2 | complex | 22 | no | simple |
| 6 | கணினி | /gaɳini/ | computer | 6 | complex | 3 | complex | 26 | no | simple |
| 7 | இன்பம் | /inbam/ | pleasure | 5 | simple | 4 | complex | 23 | no | simple |
| 8 | விமானம் | /ʋima:nam/ | flight | 7 | complex | 4 | complex | 19 | no | simple |
| 9 | நாற்காலி | /na:ɺka:li/ | chair | 7 | complex | 4 | complex | 19 | no | simple |
| 10 | உடற்பயிற்சி | /ud̪eɺpajiɺt͡ʃi/ | exercise | 11 | complex | 7 | complex | 15 | no | complex |

*Note.* ^a^An item was judged as complex if it contained a diphthong, long vowel, retroflex consonant or consonant cluster and as simple if none of these phonemic

units were present. ^b^An item was judged as complex if it contained at least one composed grapheme (built up akshara) and was otherwise considered simple. ^c^An item was judged as complex if it contained at least one pre- or suffix or represented a compound word formed of at least two root words and was otherwise considered simple.

**APPENDIX C**

**Extension of Phonological, Orthographic, and Morphological Assessment System (POMAS) by Bahr et al. (2012, 2015) for Chinese, Malay and Tamil**

**Chinese version**

| Overall error category | Language-specific error category | Further explanation | Example | |
| --- | --- | --- | --- | --- |
|  |  |  | Target | Error |
| Phonological | Phonetic radical addition, substitution or omission^a^ | A phonetical radical is a subcomponent of a character that provides a phonological clue to the pronunciation of the character. For example, the character 妈 (/ maː^1^/ - in English *mother*) is composed of the semantic radical 女 (/[nɥyː^3^](https://easypronunciation.com/)/ - in English ) on the left-hand side (**女**马) that conveys the semantic information of this character and the phonetic radical 马 (/ maː^3^/ - in English *horse*) on the right-hand side (女**马**) that provides a cue to the pronunciation of the whole character. | 妈 | 女 |
|  | Similar sounding character/word substitution^a^ | For example, same phoneme sequence, but different tone. | 写 | [些](http://hskhsk.pythonanywhere.com/cidian?q=%E4%BA%9B) |
| Graphemic-orthographic | Reconfiguration of characters or components of characters^a^ | Reconfigurations refer to reversions in the position of characters of components of characters. | 妈 | 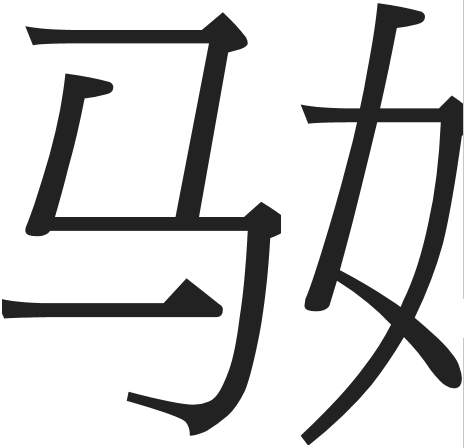 |
|  | Similar formed or structured character/grapheme substitution^a^ |  | 早 | 草 |
|  | Addition, omission or protrusion of strokes^a^ |  | 妈 | 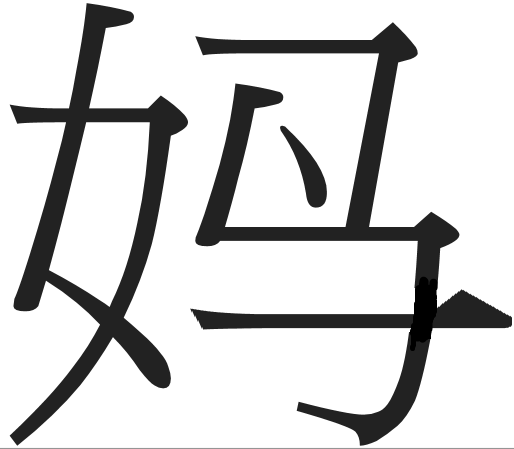 |
| Morphological-semantic | Substitution of semantically related character/word^a^ |  | 早 | 迟 |
|  | Substitution of homophone character/word^a^ | A homophone is a word that is pronounced in the same way as another word, but is spelled differently. | 坐 | 做 |
|  | Morpheme omission (character, pre-/suffix or root)^a^ | A morpheme is meaningful unit of language that cannot be further divided. In the case of Chinese we include the omission of semantic radicals or entire characters in this error category. | 妈 | 马 |
| Others | Substitution by irrelevant word/nonword | This category refers to the substitution of the target by an existing or made up word that has no semantic or phonological resemblance with the target word. | 坐 | 月 |
|  | No response |  | 写 |  |

*Note.* ^a^Directly based on Shen & Bear (2000) and Tong et al. (2009).

**Malay version**

| Overall error category | Language-specific error category | Further explanation | Example | |
| --- | --- | --- | --- | --- |
|  |  |  | Target | Error |
| Phonological | Single vowel substitution^a^ | We considered /i/, /e/, /ə/, /a/, /u/ and /o/ as Malay single vowels (Hassan, 1974; Onn, 1980: Yunus Maris, 1980). | dan | din |
|  | Single vowel addition^a^ |  | pantas | pantasa |
|  | Single vowel omission^a^ |  | ibu | bu |
|  | diphthong substitution, addition or omission^a^ | We considered /ai̯/, /au̯/ and /oi̯/ as Malay diphthongs (Hassan, 1974; Onn, 1980: Yunus Maris, 1980). | permainan | permanan |
|  | Consonant substitution^a^ |  | mata | nata |
|  | Consonant addition^a^ |  | guru | gurut |
|  | Consonant omission |  | dapur | dapu |
|  | Similar sounding word substitution |  |  |  |
|  | Partial reversal of phoneme sequence^a^ | This category was used to refer to errors in which all of the sounds of the spoken word were represented, but in an incorrect order. | membaca | memcaba |
| Graphemic-orthographic | Similar formed or structured grapheme substitution^a^ | This category included digraph errors (e.g. errors in the representation of <sy>) or similar formed single letter reversals (e.g. <b> for <d>). | dapur | bapur |
|  | Addition, omission or protrusion of strokes | This category referred to errors in the strokes needed to represent a single letter (e.g. <f> without the horizontal stroke). | televisyen | lelevisyen |
| Morphological-semantic | Substitution of semantically related word |  | pantas | lambat |
|  | Morpheme omission (pre-/suffix or root)^a^ | A morpheme is meaningful unit of language that cannot be further divided. In the case of Malay we include the omission of pre- and suffixes, as well as roots in this category. | permainan | mainan |
| Others | Substitution by irrelevant word/nonword | This category refers to the substitution of the target by an existing or made up word that has no semantic or phonological resemblance with the target word. | tidur | epal |
|  | No response |  | guru |  |

*Note.* ^a^Directly based on Bahr et al. (2012, 2015).

**Tamil version**

| Overall error category | Language-specific error category | Further Explanation | Example | |
| --- | --- | --- | --- | --- |
|  |  |  | Target | Error |
| Phonological | Single vowel substitution^a^ | We considered /i/, /i:/, /e/, /e:/, /a/, /a:/, /u/, /u:/, /o/ and /o:/ as Tamil single vowels (Schiffman, 1999). | இன்பம் | உன்பம் |
|  | Single vowel addition^a^ |  | பல | இபல |
|  | Single vowel omission^a^ |  | உடற்பயிற்சி | டற்பயிற்சி |
|  | Diphthong substitution, addition or omission^a^ | We considered /aɪ/ and /aʊ/ as Tamil diphthongs. | NA | NA |
|  | Short vowel vs. long vowel substitution^a^ | We considered /i/, /e/, /a/, /u/, /o/ as short vowels and and /i:/, /e:/, /a:/, /u:/, /o:/ as long vowels in Tamil. | கரம் | காரம் |
|  | Long vowel vs. short vowel substitution^a^ |  | நாற்காலி | நாற்கலி |
|  | Consonant substitution^a^ |  | படம் | படன் |
|  | Retroflex consonant substitution | We considered /ɳ/, /ʈ/, /ɻ/ and /ɭ/ as retroflex consonants that are easily confused for their dental and alveolar counterparts /n/, /t̪/, /r/ and /l/. | பழம் | பரம் |
|  | Consonant addition^a^ |  | பல | பலம் |
|  | Consonant omission^a^ |  | கரம் | கர |
|  | Similar sounding word substitution | This category referred to errors related to real words. | படம் | பட்டம் |
|  | Partial reversal of phoneme sequence^a^ | This category was used to refer to errors in which all of the sounds of the spoken word were represented, but in an incorrect order. | பல | லப |
| Graphemic-orthographic | Similar formed or structured grapheme substitution^a^ | This category included errors in which similarly formed or structured aksharas were confused (e.g. akshara for /ka/ vs. /sa/ or /pa/ vs. /ma/). | கரம் | சரம் |
|  | Addition, omission or protrusion of strokes | This category referred to errors in the strokes needed to represent one of the components of independent aksharas. | ப | I |
|  | Addition, omission or substitution of diacritics^a^ | This category referred to errors in the diacritics that need to be represented in a specific akshara. | கரம் | கரம |
| Morphological-semantic | Substitution of semantically related word | This category referred to real words that were related to each other conceptually. | நாற்காலி | மேசை |
|  | Morpheme omission (pre-/suffix or root)^a^ | A morpheme is meaningful unit of language that cannot be further divided. In the case of Tamil we include the omission of pre- and suffixes, as well as roots in this category. | உடற்பயிற்சி | பயிற்சி |
| Others | Substitution by irrelevant word/nonword | This category refers to the substitution of the target by an existing or made up word that has no semantic or phonological resemblance with the target word. | விமானம் | காம் |
|  | No response |  |  |  |
